# Evaluation of zinc sulfate mucoadhesive formulation on recurrent aphthous stomatitis: a randomized double-blind, placebo-controlled clinical trial

**DOI:** 10.1186/s12903-020-01194-4

**Published:** 2020-07-28

**Authors:** Anahita Ghorbani, Jafar Akbari, Maryam Boorboor, Zahra Nekoukar, Gohar Eslami

**Affiliations:** 1 Department of Oral and Maxillofacial Diseases, Faculty of Dentistry, Mazandaran University of Medical Sciences, Sari, Iran; 2 Department of Pharmaceutics, Faculty of Pharmacy, Mazandaran University of Medical Sciences, Sari, Iran; 3 Department of Clinical Pharmacy, Faculty of Pharmacy, Mazandaran University of Medical Sciences, Sari, Iran; 4 Department of Clinical Pharmacy, Faculty of Pharmacy, Cardiovascular Research Center, Mazandaran University of Medical Sciences, Sari, Iran

**Keywords:** Recurrent aphthous stomatitis, Zinc, Mucoadhesive formulation

## Abstract

**Background:**

Recurrent aphthous stomatitis (RAS) is a common lesion that affects the oral mucosa. There are several methods to treat RAS, including systemic and topical formulations. This study was conducted to evaluate the anti-inflammatory effect of topical zinc sulfate and its efficacy in the treatment of RAS.

**Methods:**

A double-blind randomized clinical trial was conducted on 46 patients with RAS. They were randomly assigned into two groups to receive a zinc sulfate mucoadhesive tablet or placebo for 7 days. The pain severity was measured at baseline and daily while the diameter of the lesion was measured at baseline and on days 3, 5, and 7. The obtained data were analyzed in SPSS V.16.

**Results:**

There was no significant difference in the mean diameter of lesions and pain at baseline between the two groups (*P* = 0.643 and *P* = 0.842, respectively). However, on the third, fifth, and seventh days of the study, the diameter of the lesion significantly reduced in the intervention group (*P* = 0.001) and the pain intensity became significantly different between groups from the fourth day of the study (*P* = 0.001).

**Conclusion:**

Zinc sulfate mucoadhesive tablet was effective in recovery and reducing the pain and diameter of the aphthous lesion and could be considered for the treatment of RAS.

**Trial registration:**

Evaluation of the effectiveness of zinc sulfate mucoadhesive tablet in the improvement of recurrent aphthous stomatitis (RAS), IRCT20151109024975N9. Registered August 1, 2018, https://en.irct.ir/trial/32423. This project was registered by the Iranian Registry of Clinical Trials (http://www.irct.ir). The IRCT ID was IRCT20151109024975N9.

## Background

One of the most common lesions in the oral cavity and mucosa is recurrent aphthous stomatitis (RAS) [1]. The origin of the term “aphtae” is the Greek word “ophthi” which implies inflammation. Because of the painful and inflammatory nature of RAS, it can decrease the quality of life and affect the daily performance of the patient [2]. The prevalence rates of RAS are 25 and 40% among adults and children, respectively [3]. Many factors, including infections, endocrine and immune system disorders, stress, genetics, nutritional deficiencies, and trauma, can cause RAS [4]. Oxidative stress also plays an important role in the formation of an aphthous lesion by damaging DNA [5]. Several methods are used to control the pain, inflammation, and infection of a mucosal lesion, including systemic therapies like vitamin B12 and folate [1], as well as topical formulations such as chlorhexidine rinse, a mouthwash of doxycycline or minocycline, lidocaine ointment and spray, and topical corticosteroids [6].

Zinc is one of the critical trace elements in humans [7]. It plays crucial roles in physiological functions, such as growth and reproduction of cells, normal functioning of the immune system, collagen synthesis, and wound healing [8, 9]. It has been proven that zinc deficiency is associated with numerous physiological defects and the pathogenesis of some oral mucosal diseases. It has been found that the administration of supplements containing zinc may improve such ulcers [10]. Although zinc deficiency among RAS patients has not been investigated yet, the delay in wound healing in zinc deficiency indicates the importance of this trace element for the metabolic responses appropriate for wound healing [7].

Deficiency of antioxidants is one of the main causes of RAS and zinc has been suggested as a useful agent for balancing cellular oxidation and reduction reactions. It is also an anti-inflammatory agent and is recommended as the sulfate salt, especially in the treatment of RAS in patients who are treated simultaneously with radiotherapy or chemotherapy [11, 12]. The efficacy of oral zinc formulation (150 mg daily) [6] and its mucoadhesive formulation (as zinc sulfate) has been reported in several clinical trials in the oral ulcer healing process [13]. A mouthwash of 5% zinc sulfate is also effective in both prophylaxis and treatment of recurrent oral ulcers [14].

Mucoadhesive tablet is a drug delivery system that attaches to the mucosal layer and provides prolonged local release of the drug from the mucosal surface. Using this medication form protects drug molecules from the first pass metabolism and a higher drug concentration is achieved on the intended location [15].

Considering the anti-inflammatory and pharmacological effects of zinc sulfate in oral ulcers and no previous study on the evaluation of zinc mucoadhesive form, this study was conducted on patients with RAS.

## Patients and methods

### Research design and ethics

The present study was a double-blind randomized placebo-controlled clinical trial (IRCT20151109024975N9) with the ethical code IR.MAZUMS.REC.1397.004. It has been approved by the Vice-Chancellor of Medical Ethics Committee of Mazandaran University of Medical Sciences on April 24, 2018.

### Participants and selection criteria

**Fig. 1:**
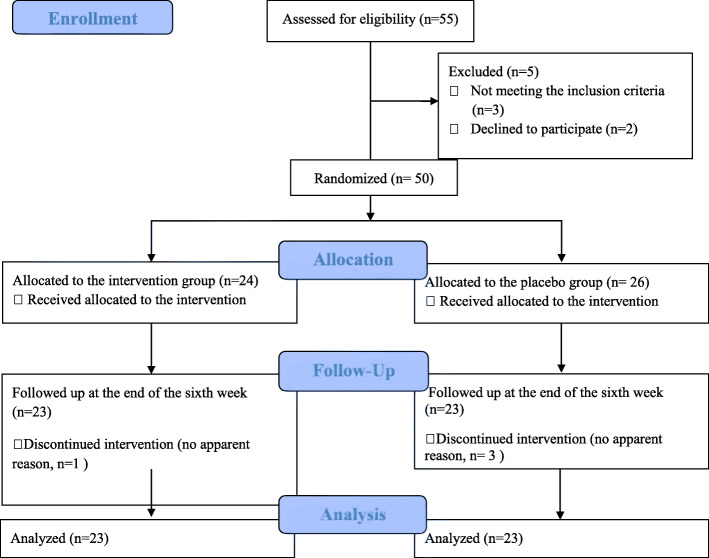
Diagram of patient enrollment and study process

As mentioned in the CONSORT (Consolidated Standards of Reporting Trials) diagram (Fig. 1), out of 55 patients (29 women and 26 men) with RAS who referred to Mazandaran Dentistry Clinic, 50 ones were selected based on the inclusion and exclusion criteria. Patients aged 16 to 45 years with a history of minor aphthous ulcers on lips or buccal mucosa were enrolled in this randomized clinical trial (RCT). The exclusion criteria were as follows: having recurrent major stomatitis and herpes simplex, having aphthous lesions on other sites than the lips and buccal mucosa, having denture, having systemic diseases (like inflammatory bowel diseases, celiac disease), taking immunosuppressive drugs during the past month or receiving antibiotics, being pregnant or lactating, and having poor health status. Also, the patients who are unable to use a mucoadhesive tablet of zinc sulfate, have syndromes that their manifestations were aphthous ulcers (like Behcet’s syndrome, use other medicines than zinc to treat aphthous lesions, smokers, and people who cannot complete the intervention for personal reasons, were excluded [16].

### Formulation preparation

Zinc sulfate (5 mg) as zinc sulfate(8H_2_O) was added to the other exepiants, including carbopol 940 and sodium alginate as formulation binding agent to the oral mucosa, starch for adjusting tablet weight (filler), and adjusting the time of the destruction of the formulation (disintegration) and eventually, sucrose to create the proper flavor and accelerate the disintegration time. These components were passed through the sieve (with mesh No. 80) and mixed and then pressed with a single punch tablet press machine [17]. ‘The placebo was the same in appearance and content as zinc sulfate mucoadhesive tablets, except for the active ingredient of zinc sulfate’.

### The study protocol

The patients were asked to visit the clinic within the first 24 h after the formation of an aphthous ulcer, and this time was considered as the baseline. Block randomization was performed by a central office using predetermined randomization lists. The patients in the intervention group received a mucoadhesive tablet of zinc, 3 times a day. The duration of the intervention was 7 days. In the placebo group, the same procedure was followed with the placebo. Tablets were supplied and participants were interviewed during the study by a dentistry student. Throughout the study, patients and the dentistry student were unaware of which intervention is being done. The patients were all informed about appropriate way of mucoadhesive tablet use. They should not drink or eat for 30 min after taking the tablets. The participants have received adequate explanations about the study protocol, possible complications and all of them signed the consent form. They were clinically examined on days 0, 3, 5, and 7, to evaluate the inflammation, the diameter of the lesions (using metal calibers) and the surrounding inflammation [18]. The patients with a lesion diameter of less than 1 mm were considered as being improved [19]. They were also trained to determine the severity of pain using the visual analog scale (VAS). This scale is a 10 cm line divided into 10 parts that 0 presents lack of pain and 10 the maximum pain. Our patients reported VAS scores in a questionnaire three times a day after their meals. It was evaluated on day 1 to 7 of the study and score of less than one was considered as pain relief.

### Data analysis

The obtained data were assessed in SPSS v.16. The distribution of data variables was analyzed by the Kolmogorov-Smirnov test and demographic characteristics were presented as mean and standard deviation. To compare the mean values before and after the intervention, the paired t test (or the Kruskal-Wallis test) was used. The independent t test (or the Mann-Whitney test) was used to compare two groups. Also, to compare the outcome of treatment, repeated measures (or Friedman) test was used in two groups. A *P* value of equal to or less than 0.05 was considered significant.

## Results

### Sample size and demographic characteristics

Out of the 55 patients referred to the Dental Clinic of Mazandaran University of Medical Sciences, three patients were excluded because of not meeting the inclusion criteria or to refuse continuing the treatment. Eventually, 46 patients (27 women and 19 men) completed the intervention (Fig. 1). According to Kolmogorov-Smirnov test results, the variables lacked a normal distribution. Thus, nonparametric tests were used to compare the variables between the two groups.

There were 13 women and 10 men in the intervention group vs 14 women and 9 men in the placebo group. The mean ± SD age of the intervention group was 38.66 ± 21.6 years and the mean ± SD age of the placebo group was 41.28 ± 24.37 years. The groups were well matched in demographic characteristics and there were no significant statistical differences between them regarding age and gender (*P* > 0.05).

### Ulcer size

**Fig. 2:**
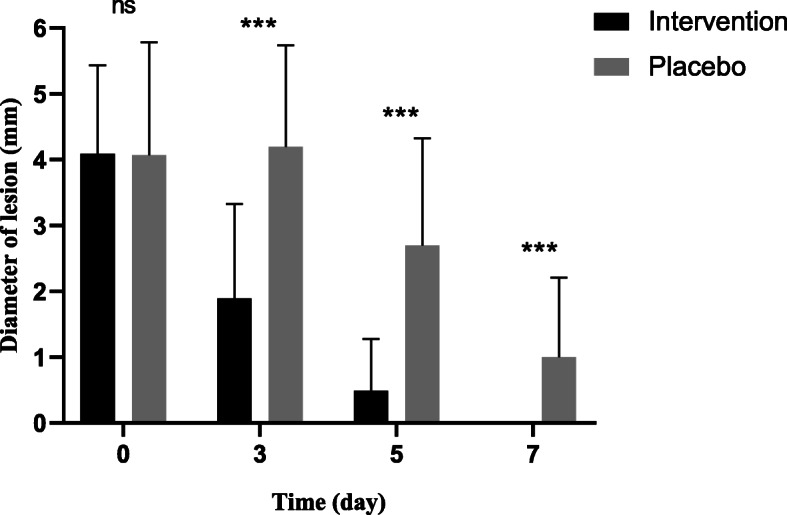
Comparing the mean diameter of the lesions between the two groups during the study. The Chi-square test (ns; not significant, * *P* ≤ 0.05, ** *P* ≤ 0.01, *** *P* ≤ 0.001)

There was no significant difference between the two groups regarding the mean diameter of the inflammatory area around the lesion on day 0 (*P* = 0.962). Based on the Chi-square test, the diameter of the lesion in the third, fifth, and seventh days of the study, was significantly (*P* = 0.001) lower in the intervention group than the placebo (Fig. 2).

### Pain intensity

**Fig. 3:**
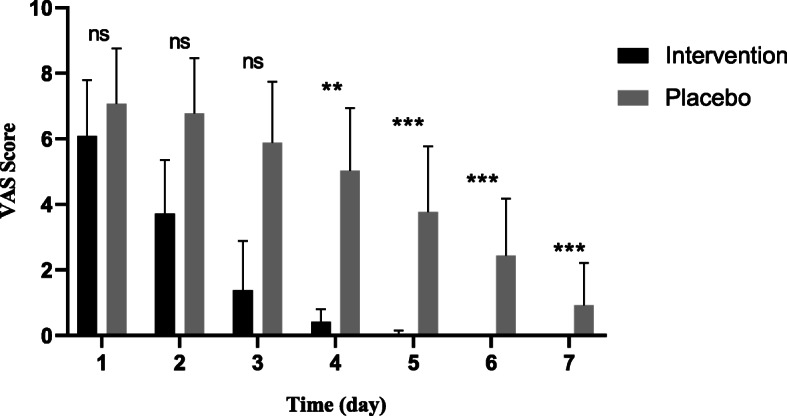
Comparing the mean VAS score between the two groups during the study. The independent t test (ns; not significant, *P ≤ 0.05, **P ≤ 0.01, *** *P* ≤ 0.001)

The VAS score was evaluated in three separate sessions every day in two groups, which to the ease of calculation, the average score of the three times was used. At baseline, there was no significant difference between groups in the level of pain (*P* = 0.842). The independent t test showed a significant difference (*P* = 0.001) between the mean VAS scores of the two groups from the fourth day of the study (Fig. 3).

### Safety assessment

No adverse effect was observed in the two groups during the study.

## Discussion

This paper was the first placebo-controlled clinical trial on using a mucoadhesive tablet of zinc sulfate in RAS. The primary outcome of this study showed that ‘the mucoadhesive formulation of zinc sulfate in patients with RAS dramatically accelerated inflammation and pain of the aphthous lesion in comparison to the placebo’. There was a significant reduction in wound diameter and its inflammation on days 3, 5, and 7 in the intervention group compared to the placebo group, which can be attributed to the anti-inflammatory and protective properties of topical zinc. On the fourth day of the study, the pain in the intervention group was significantly lower compared to the placebo group and it was resolved in the intervention group at the end of the study. No complication related to the intervention was reported during this study.

Recurrent aphthous stomatitis is a common disorder of mucosa in the oral cavity that is characterized by painful and recurrent inflammatory ulcers on the oral mucosa [20]. It has no definitive treatment because the exact etiology of RAS is still unknown [21]. The goal of treatment is to reduce the pain, duration of inflammation, and restoring normal oral function. The secondary objective is to reduce the frequency and intensity of relapse and to maintain the recovery of the disease [22]. Treatments used to improve RAS include topical anesthetics, anti-inflammatory drugs, topical corticosteroids, and local antibiotic therapy (tetracycline) [23]. Other agents like zinc supplements have also been reported in the recovery of aphthous ulcers [10]. However, based on several clinical trials, it seems that systemic zinc supplement therapy in RAS is still a matter of debate [24–26].

Contact time with mucosal tissue is important in drug delivery to the mucosa [27]. Topical zinc application leads to the regeneration of epithelial cells, reducing inflammation, and inhibition of bacterial growth [28]. Topical forms of this trace element have more predictable and effective pharmacological results in RAS remission and patients show more compliance to use such formulations [15, 29]. In the present research, the mucoadhesive tablet was placed on the aphthous wound and with its proper adhesion to the mucosa, it kept zinc in direct contact with the wound to exert its therapeutic effects. Zinc, as a micronutrient, plays a key role in proliferation regulation, immunity status, and wound healing. It adjusts DNA and RNA regeneration and renovation processes. It also controls the activities of macrophages, neutrophils, cytotoxic T cells, as well as the complement system [30]. Zinc also has antioxidant properties, including the prevention of ultraviolet radiation damage and a reduction in the risk of malignancy and is considered as an anti-inflammatory agent [11, 31–33].

Parya Haghpanah et al. reported that muco-bioadhesive containing Ginger extract was effective in the management of RAS pain but no significant impact on the size of aphthous lesions. In the present study, zinc was effective in the management of both pain intensity and the diameter of the ulcer. The sample size was also small in the mentioned study compared to our study [34].

Yanxiong Shao and Haiwen Zhou showed that reduction in ulcer size in the treated group with a mucoadhesive film containing chitosan was significantly more than that of the control group but there was no significant difference in terms of pain score, reduction in pain score and ulcer size between two groups [35]. They presented chitosan as an effective agent to use in the oral mucoadhesive film for the healing of RAS. While in this study both pain score and diameter of the lesion were lower in zinc mucoadhesive tablet group compared to the placebo group and zinc is suggested to manage RAS due to its several mechanisms like anti-inflammatory property.

Skaare et al. suggested the effectiveness of zinc and triclosan on oral ulcer healing, which was confirmed by the zinc supportive effect and the anti-inflammatory effect of triclosan [36]. In our study, because of the anti-inflammatory effect of zinc, it can be effective in the healing of aphthous ulcers.

Jahanshahi et al. designed a study to evaluate the efficacy of triamcinolone in the symptomatic treatment of RAS. The study was done by adding triamcinolone to the mucous adhesive base on 40 patients. In the intervention group, the diameter of lesion and pain in the second session were significantly lower than the placebo group (*P* = 0.043). However, no significant difference was observed in the third session (*P* > 0.05) [37]. Meanwhile, in our study by adding zinc sulfate to the mucous adhesive base, there was a significant decrease in the diameter of the lesions in the intervention group from the second to the fifth day of the study.

Since pain in the ulcer tissue is usually due to the secondary infection or mechanical and chemical stimulators [38], the use of the mucoadhesive formulation as coverage and protective agent can cause premature anesthesia and accelerate the recovery time. According to the results of the present study, zinc can reduce the pain, diameter of the wound, its surrounding inflammation, and accelerates the recovery time of RAS. ‘The limitation of this project was that the patients were not directly monitored for right dose consumption’. It is recommended that a multi-center study be conducted in the future to gain more reliable results.

## Conclusion

This study demonstrated statistically and clinically the effectiveness of zinc mucoadhesive tablets as a topical drug delivery system, on reducing pain, the diameter of the wound, and duration of the recovery period of RAS compared with the placebo group.

## Data Availability

The datasets used and or analyzed during the current study are available from the corresponding author on reasonable request.

## Abbreviations

RAS’: Recurrent Aphthous Stomatitis
SPSS: Statistical Package for the Social Sciences
RCT: Randomized Clinical Trial
VAS: Visual Analog Scale
SD: Standard Deviation
UV: Ultra Violet
